# Risk factors for early mortality after hepatectomy for hepatocellular carcinoma

**DOI:** 10.1097/MD.0000000000005028

**Published:** 2016-09-30

**Authors:** Chao-Wei Lee, Hsin-I Tsai, Chang-Mu Sung, Chun-Wei Chen, Shu-Wei Huang, Wen-Juei Jeng, Tsung-Han Wu, Kun-Ming Chan, Ming-Chin Yu, Wei-Chen Lee, Miin-Fu Chen

**Affiliations:** a Department of Surgery, Chang Gung Memorial Hospital, Linkou; b College of Medicine, Chang Gung University, Guishan, Taoyuan; c Graduate Institute of Clinical Medical Sciences, Chang Gung University, Guishan, Taoyuan; d Department of Anesthesiology,Chang Gung Memorial Hospital, Linkou; e Department of Gastroenterology and Hepatology, Chang Gung Memorial Hospital, Linkou, Taiwan (R.O.C.).

**Keywords:** hepatectomy, hepatocellular carcinoma, hepatoma, liver resection, mortality, RAM score, risk factors

## Abstract

Despite advances in surgical technique and medical care, liver resection for hepatocellular carcinoma (HCC) remains a high-risk major operation. The present study evaluated the risk factors for early mortality after hepatectomy.

We retrospectively reviewed records of patients undergoing liver resection for HCC between 1983 and 2015. A point score (Risk Assessment for early Mortality (RAM) score) for hepatectomy was developed based on multivariate analyses.

Three hundred eighty-three patients (11.3%) expired within 6 months after the operation. Logistic regression analyses identified that operative duration >270 minutes and blood loss >800 cc were significant predictors of major surgical complications (*P* = 0.013 and 0.002, respectively). On the other hand, diabetes mellitus, albumin ≤3.5 g/dL, α-fetoprotein (AFP) >200 ng/mL, major surgical procedure, blood loss >800 cc, and major surgical complications were independent risk factors for early mortality after hepatectomy (*P* = 0.019, <0.001, <0.001, 0.006, 0.018, and <0.001, respectively). Risk Assessment for early Mortality score (RAM score) identified 3 subgroups of patients with distinct 6-month mortality rate, with Class III (score 10) having highest risk of early mortality.

Our study demonstrated that meticulous surgical techniques to minimize blood loss and avoid prolonged operative time may help decrease the occurrence of major surgical complications. In addition to major surgical complications, diabetes mellitus, hypoalbuminemia, high AFP, massive blood loss, and major surgical procedure are also associated with early mortality after liver resection. Further study is warranted to validate the utility of RAM score as a bedside scoring system to predict postoperative outcome.

## Abstract 2

Supplemental Digital Content is available in the text

## 1 Introduction

Hepatocellular carcinoma (HCC) is the most common primary malignancy of the liver with an estimated annual death incidence of approximately 700,000 worldwide.^[1]^ In Taiwan, it is the second most common cause of cancer death and causes more than 8000 deaths each year.^[2]^ Surgical resection remains the most effective therapy in selected patients, but the coexisting underlying liver diseases, such as chronic hepatitis B or C and alcoholic liver disease, had limited the extent and feasibility of liver resection. Earlier before 1980s, liver resections in the presence of liver cirrhosis was associated with a relatively high mortality rate in the range of 10% to 30%, and were therefore largely limited to minor resections.^[3–10]^ With improvements in patient selection, surgical techniques, and postoperative care, the mortality rate has improved dramatically in recent decades.^[3,11–13]^ The unexpected occurrence of death after the operation, however, is still catastrophic to both the patients’ family and surgeon. As a result, the identification of potential risk factors for mortality before operation is of paramount importance. Previous studies had demonstrated multiple risk factors for perioperative morbidity and mortality after liver resection^[3,12,14]^; nevertheless, the study end-point in most series was set at 30 days after the operation. Since many patients could survive the first postoperative month but still suffered from mortality several months after the operation, the aforementioned recognized risk factors may be less applicable. The purpose of this study was therefore to unravel the potential risk factors for in-hospital and 6-month mortality and propose a scoring system in an attempt to predict postoperative outcome and risk of early mortality immediately after the operation.

## 2 Materials and methods

### 2.1 Patients

From January 1983 to January 2015, records of patients with histologically proven primary HCC from the Cancer Registry of the Cancer Center, Chang Gung Memorial Hospital, Linkou, Taiwan were retrospectively reviewed. Only patients who underwent curative hepatectomy by our surgical team were included in the study. Patients who underwent only exploratory laparotomy for liver tumor biopsy, who had multiple distant metastases before operation, who did not have detailed preoperative/intraoperative clinical records, or who did not have regular postoperative out-patient follow-up were excluded from our study. A total of 3383 patients were evaluated eventually, and their clinicopathological data were retrieved from the prospectively collected database. Our primary study endpoints were risk factors for major complication and 6-month (early) mortality. The RAM (Risk Assessment for early Mortality) score was established based on risk factors for 6-month mortality. The secondary endpoints were risk factors for 30-day and in-hospital mortality. The study end date was December 31, 2015, and tumor staging was based on the American Joint Committee on Cancer (AJCC) TNM staging system for HCC.^[15]^ This study was approved by the Institutional Review Boards of Chang Gung Memorial Hospital (CGMH IRB No: 201600359B0).

### 2.2 Preoperative assessment, surgical technique, and postoperative management

Preoperative diagnosis of HCC was established by characteristic features on imaging by either triphasic computed tomography (CT), magnetic resonance imaging (MRI), hepatic arteriography, and/or a serum α-fetoprotein (AFP) level greater than 200 ng/mL. Resection criteria were constant over the entire study period, including a lack of cancerous thrombi in the main trunk of the portal vein, no distant metastasis to other organs, a technically operable main tumor in the preoperative evaluation, and a adequate liver functional reserve. Liver function was routinely assessed preoperatively by Child–Pugh classification and indocyanine green retention test. A previous study identified an indocyanine green retention at 15 minutes (ICG-15) of less than 14% as the safety limit for major hepatic resection.^[16]^ In our institute, an ICG-15 ≤10% was the prerequisite for major hepatic resection. On the other hand, in patients with higher ICG-15, extensive hepatectomy could also be performed if the liver biochemistry was satisfactory and the size of the future liver remnant was considered adequate according to preoperative CT and intraoperative assessment.^[17]^

All operations were performed by experienced hepatobiliary surgeons in the same surgical department. In all patients, intraoperative exploration by both manual palpation and ultrasonography was conducted to define the extent of the tumor(s), any invasion of the portal or hepatic veins, the texture of liver parenchyma, and the size of future liver remnant. Inflow control, which included Pringle maneuver, Glissonian pedicle control,^[18,19]^ selective vascular control, and total vascular exclusion, was applied whenever necessary according to individual surgeon's discretion. Parenchymal transection was performed by using either crush clamp technique, ultrasonic dissector, or other energy device based on surgeon's preference. Hemostasis was achieved and bile leakage was repaired meticulously in each operation.

Patients were monitored and cared postoperatively in either intensive care unit (ICU) or general wards depending on individual patient's condition. The duration of ICU stay depended on individual patient's clinical condition. Intravenous crystalloid fluids were given to maintain fluid requirement. First line broad-spectrum antibiotics were administered intravenously for 3 to 7 days in all patients. Fresh frozen plasma or albumin was given if the plasma albumin level was lower than 3.0 g/dL. Hemogram and biochemical liver function tests were examined 2 and 7 days after liver resection. Oral feeding or enteral nutrition was encouraged and resumed as early as possible after operation. Parenteral nutrition was provided if patient was malnourished or enteral nutrition could not be resumed 5 to 7 days after operation. All patients received blood tests and triphasic CT examination 1 to 2 months after operation. Out-patient follow-up with serial lab tests and image study was arranged every 2 to 3 months after hospital discharge.

### 2.3 Definition

Preoperative, intraoperative, and postoperative data were retrieved from a prospectively collected database. Major liver resection defined resection of three or more liver segments.^[20]^ Major surgical complications comprised grade III and grade IV surgical complications,^[21]^ which included postoperative bleeding requiring angiographic embolization or reoperation, major biliary complications requiring drainage or endoscopic intervention, intestinal obstruction requiring operation, upper gastrointestinal bleeding requiring endoscopic hemostasis, massive ascites or pleural effusion requiring paracentesis, sepsis of any etiology, liver failure, renal failure, respiratory failure, or any condition dictating ICU care. Thirty-day mortality was defined as the occurrence of death within 30 days after the operation. In-hospital mortality was defined as death during the same hospital stay, and 6-month, or early mortality was defined as the occurrence of death within 6 months after the operation. The cause of early mortality included HCC recurrence/metastasis, hepatic failure due to liver cirrhosis, and postoperative surgical complications. Overall survival (OS) was defined by the time elapsing from the date of diagnosis to either the date of death or the date of the last contact.

### 2.4 Statistical analysis

The statistical analysis was performed with IBM SPSS Statistics Version 21 for Windows (IBM Corporation, NY). Fisher exact test or Pearson *χ*^2^ test was used to analyze categorical data. Student *t* test was used to analyze continuous variables. Statistical significance was defined as *P* values < 0.05 in 2-sided tests. Significant variables in the univariate analysis were then subjected into a stepwise logistic regression analysis (conditional forward selection) as candidate variables. The regression coefficients of the independent variables identified by logistic regression model were multiplied by two and rounded to integer in order to calculate the Risk Assessment for early Mortality score (RAM score). The influence of the RAM score on OS and early mortality was then examined by Kaplan–Meier analysis and Log rank test.

## 3 Results

### 3.1 Patient demographics and operative variables

A total of 3386 patients with HCC underwent liver resection during the study period. The median follow-up time was 39.3 months. Their demographic and clinical data are summarized in Table 1. Among patients who did not receive liver resection immediately after diagnosis, transarterial chemoembolization (TACE) was the most common preoperative treatment (254 patients, 92.7%), followed by percutaneous ethanol injection (3.8%) and radiofrequency ablation (2.9%). Major resection was conducted in 1348 patients (41.6%), with trisegmentectomy being the most common major resection procedure (14.8%), followed by right hepatectomy (13.9%), left hepatectomy (7.1%), extended right hepatectomy (4.1%), and extended left hepatectomy (1.6%). Pringle maneuver (87.7% of all inflow control) remained the most common form of hepatic inflow control during operation.

**Table 1:**
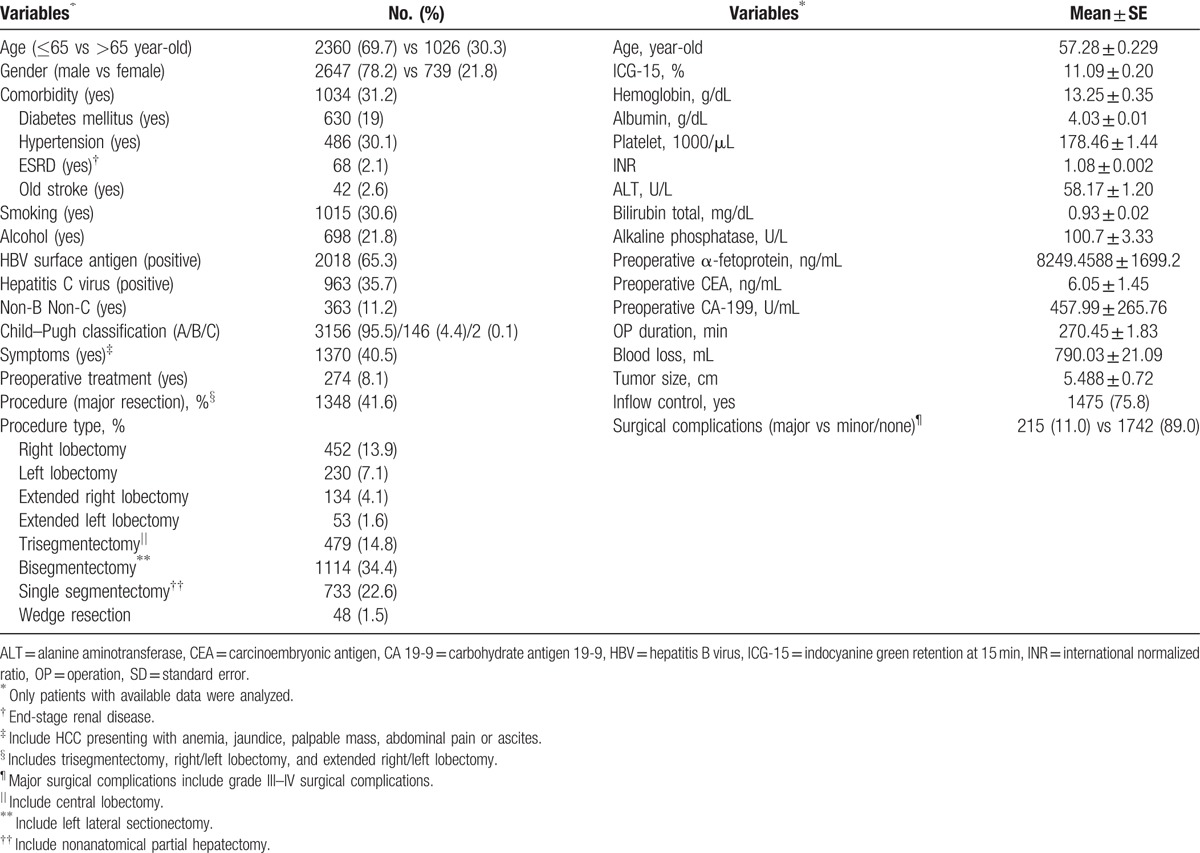
Demographic data of patients with hepatocellular carcinoma undergoing hepatectomy (n = 3386).

### 3.2 Risk factors of major surgical complications

Among 3386 patients, 1957 had detailed records of their postoperative complications and were analyzed. Among them, 1250 patients (63.9%) were either uneventful or experiencing only grade I complications during postoperative period. Grade II complications occurred in 491 patients (25.1%) and they can be treated with pharmacological therapy, parenteral nutrition, or blood transfusion. Grade III complications happened in 147 patients (7.5%), and these patients required either surgical, endoscopic, or radiological interventions. Life-threatening, or grade IV complications developed in 68 patients (3.4%), and intensive critical care with organ support systems were employed in these patients.

For risk factor analysis, statistical analysis was conducted and the results are summarized in Table 2. Eighteen preoperative variables and 4 operative variables that may potentially affect the operative outcome were included in the initial univariate analysis. Variables with a *P*-value less than 0.05 by univariate analysis were subjected to a stepwise multivariate logistic regression analysis. The result showed that operative duration greater than 270 minutes (hazard ration (HR) 1.946; *P* = 0.013) and blood loss greater than 800 mL (HR 2.299; *P* = 0.002) were 2 independent risk factors for the occurrence of major surgical complications after hepatectomy.

**Table 2:**
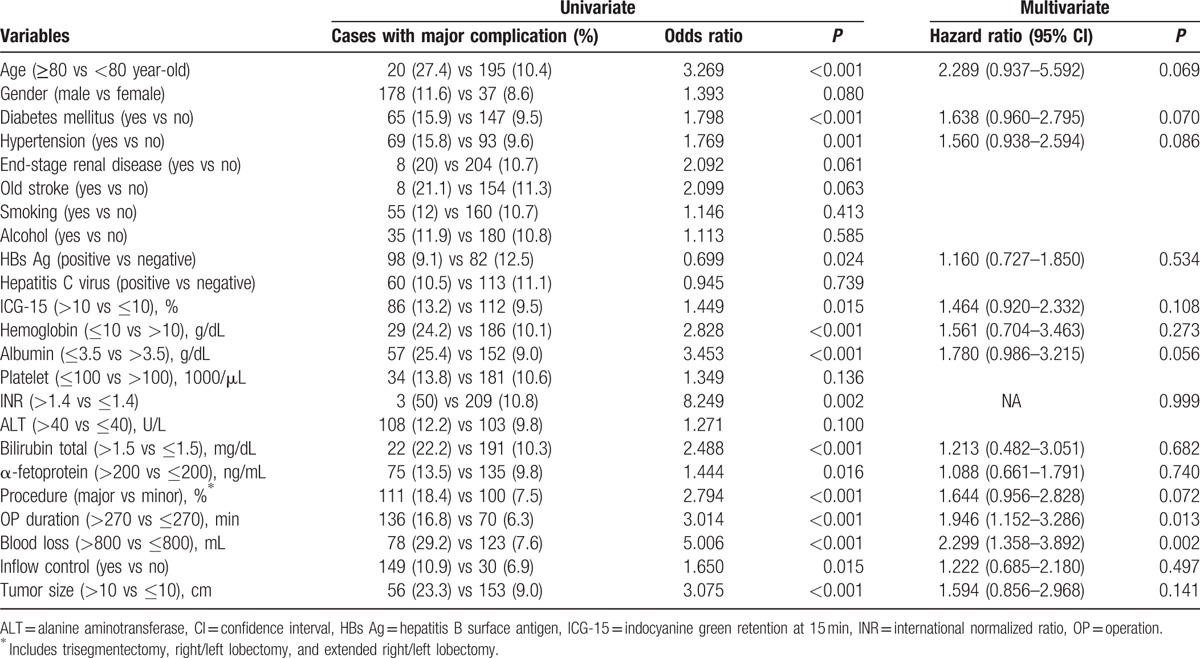
Univariate and multivariate analyses of risks factors for major complications after hepatectomy for hepatocellular carcinoma (n = 1957).

### 3.3 Risk factors for 30-day and in-hospital mortality

Sixty-one patients (1.8%) died within 30 days after hepatectomy. The mean hospital stay for these patients was 12.5 days (range 0–30 days). Statistical analysis of 18 preoperative variables and 6 operative variables was conducted and is summarized in Table 3. On the other hand, 97 patients (2.9%) died during the same hospitalization of hepatectomy. The mean length of survival of these patients was 24.5 days (range 0–123 days). Risk factors for in-hospital mortality were analyzed and are summarized in Table 4.

**Table 3:**
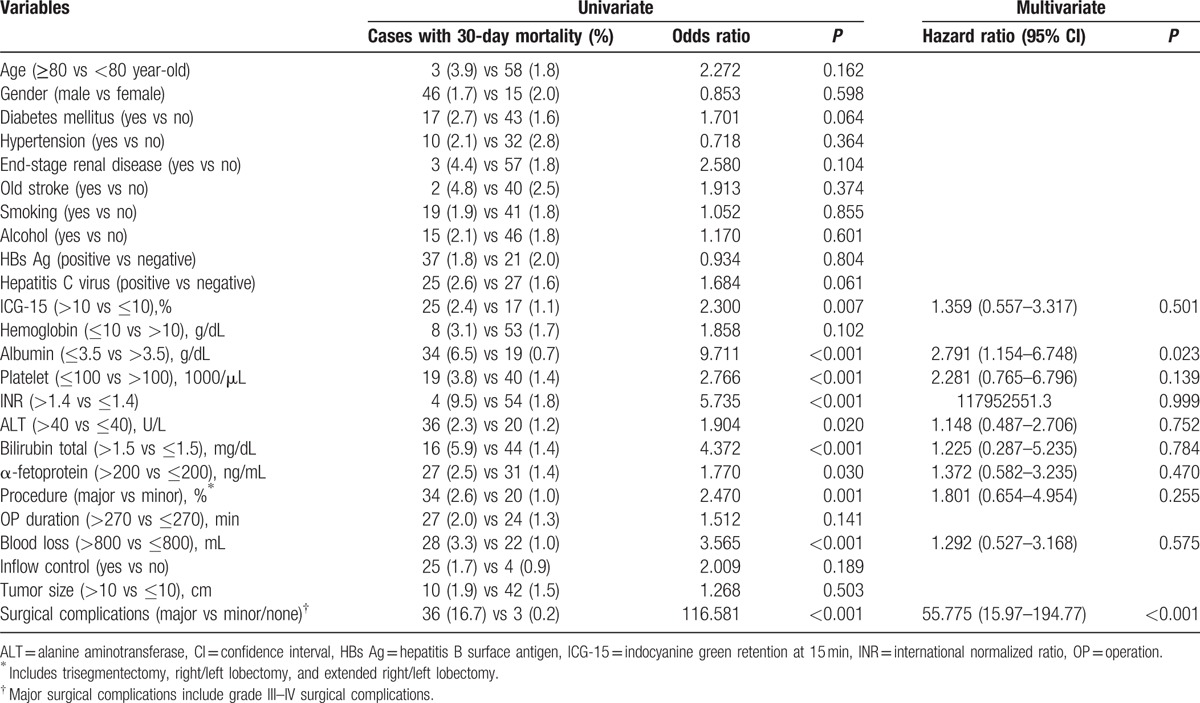
Univariate and multivariate analyses of risks factors for 30-day mortality after hepatectomy for hepatocellular carcinoma.

**Table 4:**
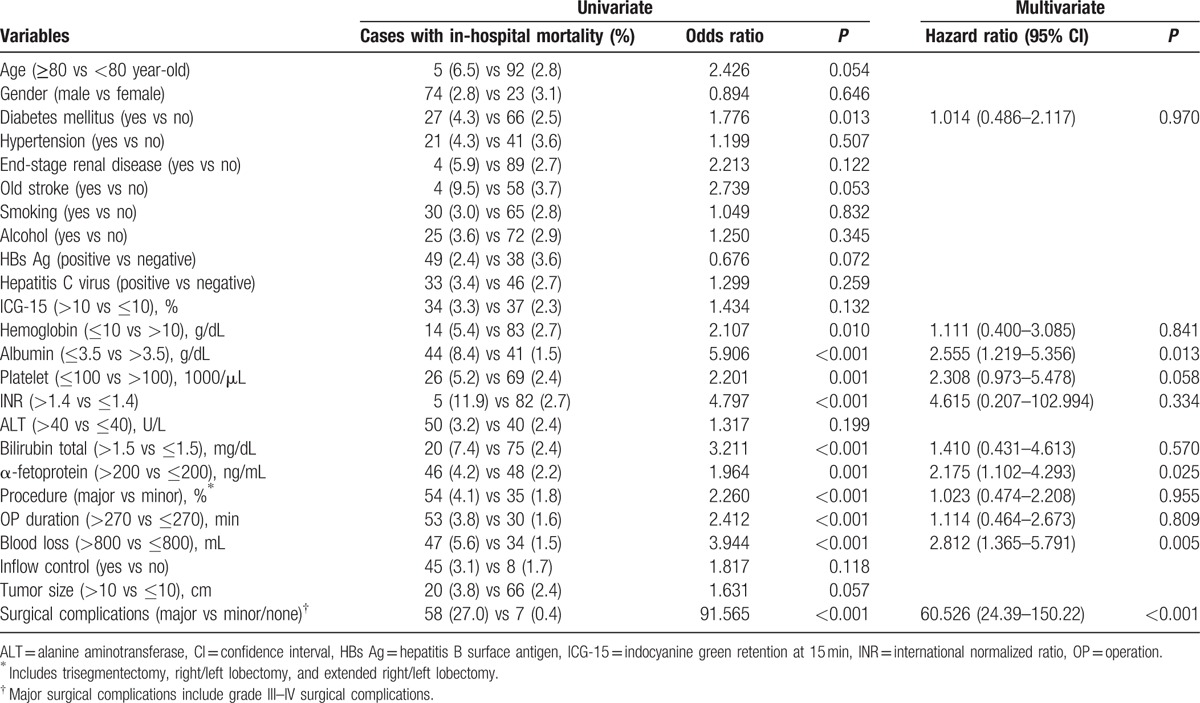
Univariate and multivariate analyses of risks factors for in-hospital mortality after hepatectomy for hepatocellular carcinoma.

### 3.4 Risk factors for 6-month (early) mortality

Three hundred eighty-three patients (11.3%) died within 6 months after hepatectomy. The mean survival of these patients after operation was 81 days (range 0–179 days). Their risk factor analysis is summarized in Table 5. The significant risk factors identified by univariate analysis were subjected to a stepwise multivariate logistic regression analysis. The result showed that diabetes mellitus (HR 1.743; *P* = 0.019), albumin ≤3.5 g/dL (HR 2.998; *P* < 0.001), AFP >200 ng/dL (HR 2.731; *P* < 0.001), major surgical procedure (HR 2.014; *P* = 0.006), blood loss >800 mL (HR 1.874; *P* = 0.018), and major surgical complications (HR 5.522; *P* < 0.001) were 6 independent risk factors for the occurrence of mortality within 6 months after hepatectomy for HCC.

**Table 5:**
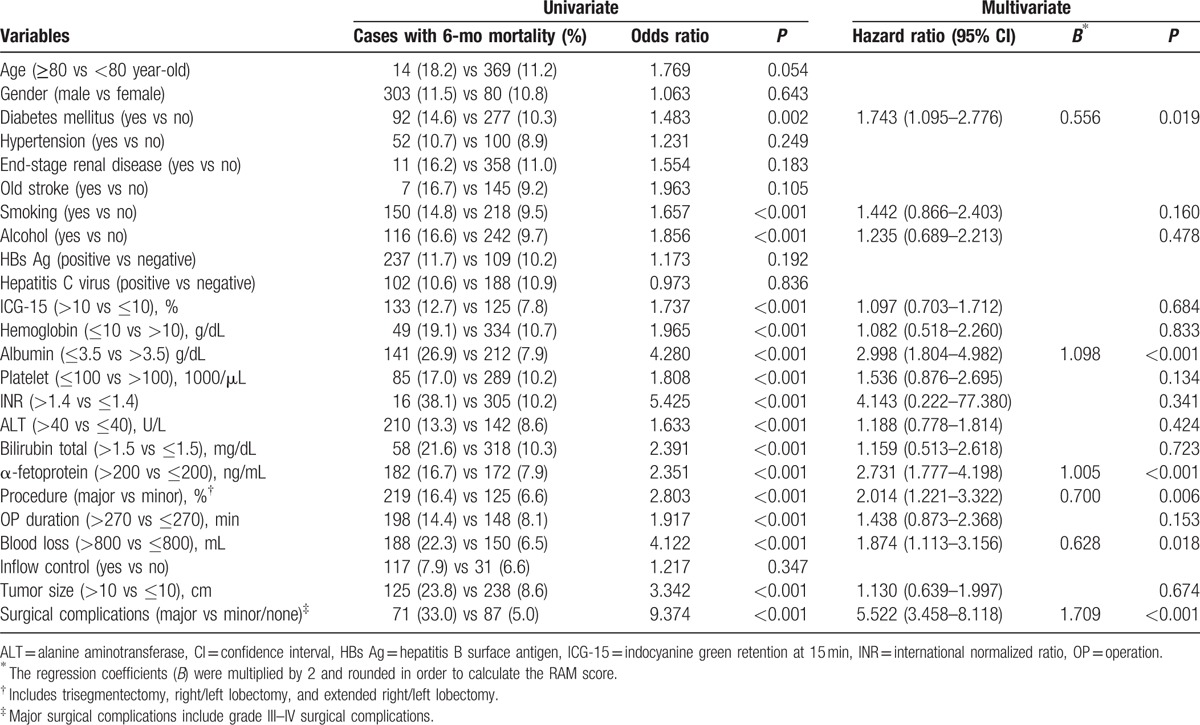
Univariate and multivariate analyses of risks factors for 6-month mortality after hepatectomy for hepatocellular carcinoma.

### 3.5 The development of RAM score

The respective calculated regression coefficient (*B*-value) of the 6 independent risk factors was multiplied by 2 and rounded to integer in order to formulate a scoring system predictive of early mortality (6-month mortality) after hepatectomy. The “Risk Assessment for early Mortality (RAM)” score for hepatectomy for HCC was developed (Table 6).

**Table 6:**
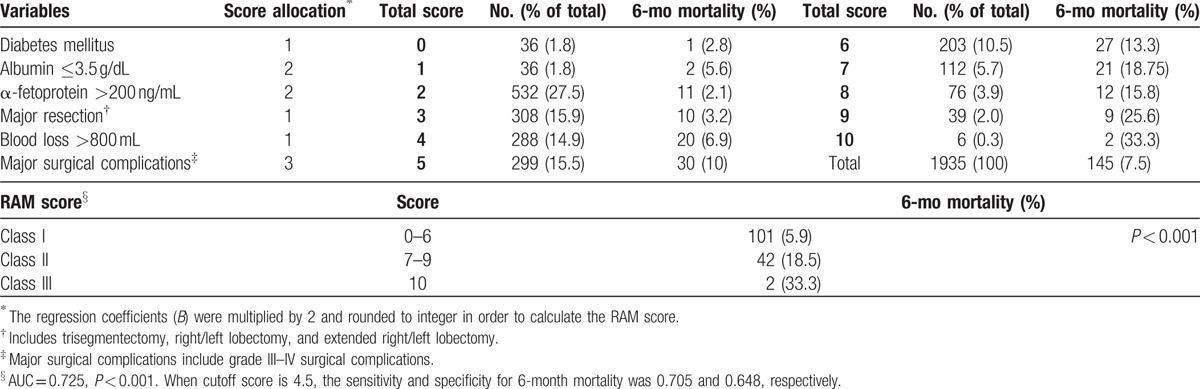
Risk Assessment for early Mortality (RAM) score for hepatectomy for hepatocellular carcinoma.

**Figure 1:**
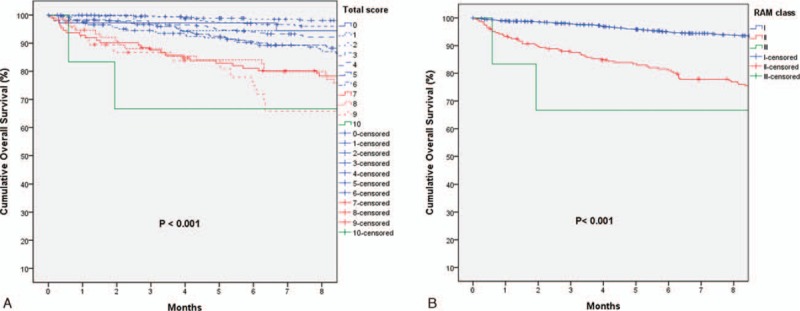
(A and B) Six-month Kaplan–Meier survival curves and predictive significance of the RAM score. (A) Predictive significance of the single point scores. The higher the individual RAM score, the higher the risk of 6-month mortality after hepatectomy for HCC. The development of a trichotomized RAM score was achieved by visual inspection of the Kaplan–Meier survival curves. Three groups of patients with distinct 6-month survival were identified, with score 0 to 6, 7 to 9, and 10 as 3 different groups. (B) Predictive significance of the RAM class. RAM class I had only 6% risk of early mortality, while one-third of patients died within 6 months after hepatectomy if they were RAM class III.

The RAM score was the summation of scores of 6 independent variables. Only patients (1935 patients) with complete information of all 6 variables had their RAM score calculated. Among them, 145 patients died within 6 months after operation. As shown in Table 6, the RAM score was significantly associated with 6-month mortality, with higher score indicating higher risk of early mortality (AUC 0.725, *P* < 0.001). Their respective 6-month survival curve is illustrated in Fig. 1A. After visual inspection of the Kaplan–Meier curves, 3 groups of patients with distinct early mortality rates were identified, with score 0 to 6, 7 to 9, and 10 as 3 different groups. These 3 group of patients were then designated as RAM class I, II, and III, respectively, and their 6-month survival curves are illustrated in Fig. 1B. As shown in Table 6 and Fig. 1B, RAM class I had only 6% risk of early mortality, while one-third of patients died within 6 months if they were RAM class III. In addition to early mortality, the RAM score was also significantly associated with OS. The OS curves are illustrated in Supplemental Figure 1. Furthermore, the RAM score was still predictive of OS even if we excluded patients with in-hospital mortality (Supplemental Figure 2).

## 4 Discussion

Despite improvements in surgical technique, operative instruments, and postoperative care, liver resection still carries substantial risks in modern era. Recent studies reported perioperative mortality rates of 2.6% to 8.4%^[3,12,22,23]^ for HCC patients undergoing major liver resection. The definition of perioperative mortality in most published studies was death in hospital or death within 30 days after the operation. In the present study, the 30-day mortality rate was 1.8% and in-hospital mortality rate was 2.9%, which were comparable to those of previous studies. The 1.1% difference between in-hospital mortality rate and 30-day mortality rate in our study may indicate that even if the patient could live for more than 30 days postoperatively, death could still occur 1 month after the operation. Likewise, some patients, even after discharge, required frequent emergency room (ER) visits or rehospitalization to deal with postoperative complications. And still several of these patients eventually expired several months after the operation without evidence of tumor recurrence. To minimize medical costs and optimize surgical outcome, it is of paramount significance to clarify significant risk factors contributing to major postoperative complications and early mortality after liver resection for HCC. Our study, to the present date, is the only research that analyzed the surgical outcome from 1 month to 6 months after the operation. With more than 3300 patients operated in a single institute, our study is also by far one of the largest reports dealing with the surgical results in the English literature.

The surgical complication rate may differ among literatures due to different definition or criteria. An overall complication rate of 24% to 70% was reported in published studies.^[3,12,14,22–24]^ In our study, 36% of patients suffered from grade II or more surgical complications, which was comparable to most of the published series. Since the most common postoperative complications after liver resection for HCC were ascites and pleural effusion,^[3,22,25]^ which, although a nuisance for surgeons and patients, could mostly be controlled by pharmacotherapy and were rarely life-threatening, it may be more practical clinically to analyze the risk factors for significant or life-threatening complications. In present study, the incidence of major surgical complications were 10.9%, which was comparable to other published studies.^[3,26]^

It was reported that preoperative platelet count, serum aspartate aminotransferase (AST)/alanine aminotransferase (ALT) level, intraoperative blood loss, portal clamping, perioperative blood transfusion, comorbid illness, Child–Pugh classification, ICG retention test, additional procedures, the American Society of Anaesthesiology (ASA) score, extent of resection, and presence of hepatic steatosis were risk factors for the occurrence of postoperative complications after liver resection for HCC.^[3,12,23–26]^ Because most (96%) patients in our study were Child–Pugh A, the impact of Child–Pugh classification on the occurrence of major complications may be biased and underestimated; as a result, we decided not to include this parameter into our final analysis. We found in the present study that operative duration greater than 270 minutes and blood loss greater than 800 mL were 2 independent risk factors for the occurrence of major surgical complications after hepatectomy. Old age, comorbidity such as diabetes mellitus or hypertension, high ICG-15, major procedure, anemia, hypoalbuminemia, coagulopathy, and large tumor size were not independently related to major complications. Our results indicated that even for patients with suboptimal preoperative clinical data, surgeons can still conduct liver resections as long as we extreme our every effort to minimize blood loss and shorten operative duration. Massive blood loss often required blood transfusions, which had been shown to be associated with adverse effects on the immune system, leading to an increased risk of postoperative infection.^[27]^ Furthermore, prolonged abdominal operations have been proved to be at higher risk for surgical site infections.^[28]^ All these studies supported that liver surgeons should try to reduce blood loss and operative duration by employing any form of vascular control, vessel-sealing device, and parenchymal transection technique during hepatectomy. Since inflow control was not a significant risk factor for major surgical complications in present study, and previous research showed that Pringle maneuver during liver transection resulted in less blood loss,^[29]^ we believe that the use of portal clamping in selected patients is justified. However, our study did not suggest liberal performance of liver resections in patients with poor liver function and/or reserve. Since all of our patients were Child–Pugh A or early B, ECOG 0, and had adequate future liver remnant, we believe that prudent patient selection and comprehensive preoperative preparation may be as important as excellent surgical technique in terms of surgical outcome.

As for risk factors for mortality, we found in the present study that, regardless of study end point, albumin ≤3.5 g/dL and major surgical complications were 2 independent risk factors for the occurrence of mortality. The pathological variables (i.e., vascular invasion, daughter nodules, etc.) were not related to 6-month mortality (data not shown). Massive blood loss and high AFP, although not significant at 30-day study point, became significantly important when study endpoint was in-hospital or 6-month mortality. Likewise, diabetes mellitus and major surgical procedure became independent risk factors for mortality when endpoint was set at 6 months. Since diabetes mellitus, procedural type, and AFP level are essentially inherent to the patient or tumor, careful patient selection, adequate preoperative nutritional support to restore albumin, and meticulous surgical technique to avoid massive blood loss and major complications were of paramount importance for liver resections for HCC as a result.

In order to predict the short-term outcome of hepatectomy, we proposed the RAM scoring system by incorporating the significant risk factors of 6-month mortality. The results turned out that the higher the RAM score/class, the higher the risk of 6-month mortality. In addition, the RAM score was significantly associated with OS, indicating that the perioperative variables would also influence the long-term oncological outcome and should not be overlooked. Indeed, massive blood loss usually required blood transfusion, and it was reported that blood transfusion was an independent adverse prognostic factor of long-term disease-free and OS after hepatectomy.^[30]^ By minimizing blood loss and avoiding major complications, we surgeons could improve surgical as well as oncological outcomes as a result.

In addition to patient and tumor factors, viral factors (hepatitis B virus (HBV) or hepatitis C virus (HCV)) also influenced the treatment strategy and outcome of HCC patients. Previous studies have shown that high baseline HBV viral load was associated with shorter disease-free survival (DFS) and OS after hepatectomy, and adequate antiviral therapy after hepatectomy could prolong both DFS and OS.^[31–35]^ The RAM score as a result, should take either viral load factor or antiviral therapy into consideration. Nevertheless, many of our patients could not receive either viral load examination or antiviral therapy during the study period, these 2 important factors were not incorporated into our final analysis. Further study regarding viral factors is thus warranted to more precisely predict patient outcome after operation.

The RAM score is clinically relevant for several reasons. First, it is simple and easily applicable in a real-life clinical setting without requirement of massive calculations. Second, the classification can inform the surgeon and patient the likelihood of early mortality after hepatectomy for HCC. Third, it can exclude patients from risky operations if the preoperative RAM score is already high. Last but not the least, it can help identify which patients may require more intensive postoperative care and should not be discharged as usual routine. The RAM score, as a result, can be applied to clinical practice to predict the outcome in the perioperative period.

However, since our study is a retrospective analysis based on clinical data from a single institute, incomplete data were inevitable when reviewing records from a very long time ago. The lack of validation group is another flaw of present study. Further research by either an external cohort or prospective trial is thus warranted to validate the findings of our study.

In conclusion, our study demonstrated that meticulous surgical techniques to minimize blood loss and avoid prolonged operative time may help decrease the occurrence of major surgical complications after hepatectomy. In addition to major surgical complications, we should aware that diabetes mellitus, hypoalbuminemia, high AFP, massive blood loss, and major surgical procedure are independently associated with early mortality for patients undergoing liver resection. RAM score is an effective beside tool to predict the short-term outcome immediately after hepatectomy. Further study by either an external cohort or prospective trial is warranted to validate the utility of RAM score as a predicting tool for postoperative outcome.

## Acknowledgment

We are grateful to all our colleagues and authors in the Department of Cancer Center, Department of Gastroenterology and Hepatology, Department of Surgery, Department of Anesthesiology, and Chang Gung University for their technical assistance.

## Supplementary Material

Supplemental Digital Content
