# Supplementary material for: Risk factors for early mortality after hepatectomy for hepatocellular carcinoma
Source: Medicine (Baltimore). 2016 Sep 30;95(39):e5028. doi: 10.1097/MD.0000000000005028 (PMC5265968; doi:10.1097/MD.0000000000005028)

**Supplemental Information**

**Supplemental Figure Legends**

**Supplemental Figure 1. (A-B) Kaplan-Meier OS curves and predictive significance of the RAM score. (A)** Long-term prognostic significance of the single point scores. The higher the individual RAM score, the worse the long-term overall survival after liver resection for HCC. **(B)** Long-term prognostic significance of the RAM class. The RAM class I patients enjoyed a median OS of 144.9 months (95% CI 104.7 ~ 185.1 months), the RAM class II patients had a median OS of 51.8 months (95% CI 33.0 ~ 70.5 months), while the RAM class III patients had a median OS of only 9.3 months (95% CI 0 ~ 23.6 months)(*P*░<░0.001).


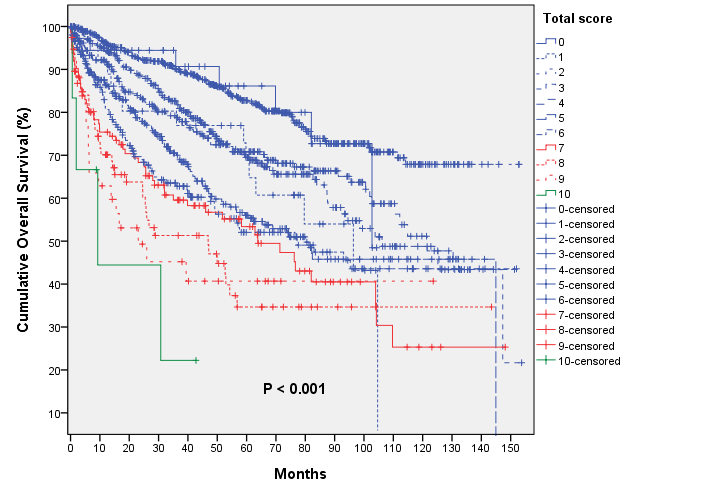


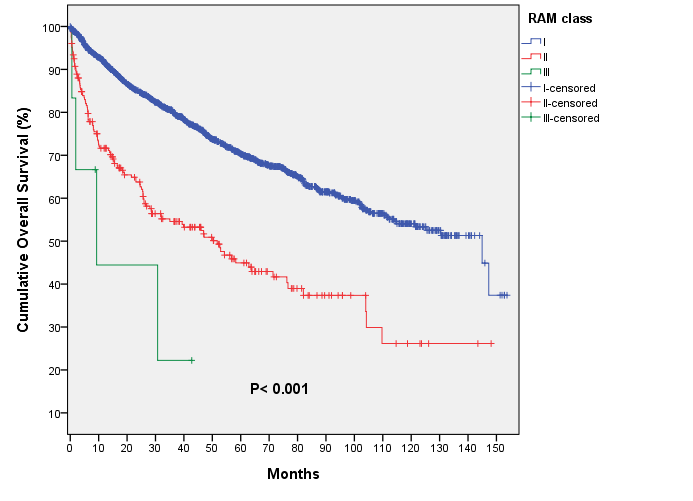


**Supplemental Figure 2. (A-B) Kaplan-Meier curves excluding in-hospital mortality.** The RAM score (A) and RAM class (B) were still predictive of overall survival after hepatectomy if we excluded patients with in-hospital mortality.


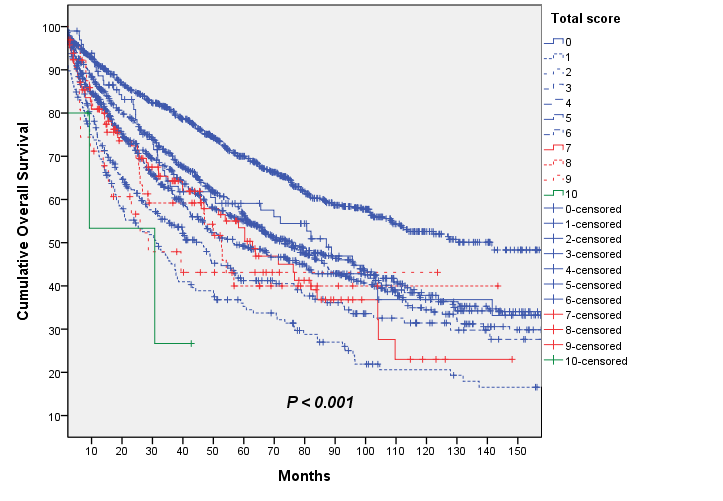


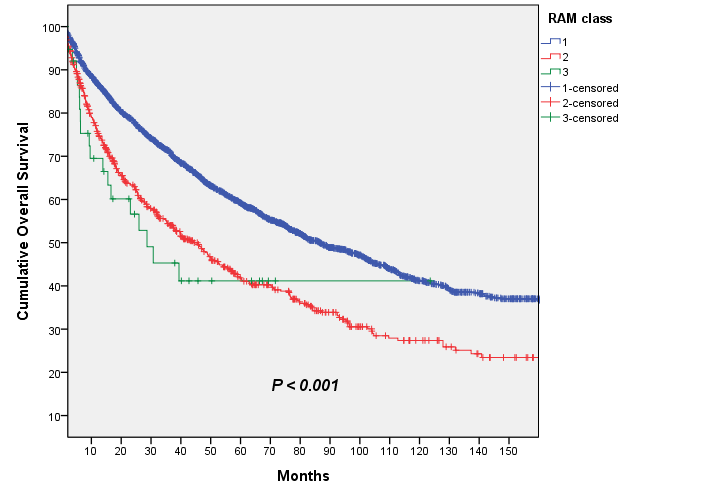

Supplement: Supplemental Digital Content [file medi-95-e5028-s001.doc]
